# Supplementary material for: Alpha-Glucosidase Inhibitory Diterpenes from Euphorbia antiquorum Growing in Vietnam
Source: Molecules. 2021 Apr 13;26(8):2257. doi: 10.3390/molecules26082257 (PMC8069799; doi:10.3390/molecules26082257)
Supplement: Supplementary file 1 [file molecules-26-02257-s001.pdf]

ALPHA-GLUCOSIDASE INHIBITORY DITERPENES FROM *EUPHORBIA ANTIQUORUM* GROWING IN VIETNAM

Table S1. ^{1}H-NMR data for compounds **1-4** in CD_3OD.

No	**1**	**2**	**3**	**4**
	δ_H ppm, *J* (Hz)	δ_H ppm, *J* (Hz)	δ_H ppm, *J* (Hz)	δ_H ppm, *J* (Hz)
1	3.52 t (3.2)	2.18 m<br>1.56 m	1.85 m<br>1.42 m	1.35 m<br>1.25 m
2	2.24 dt (2.8,15.1)<br>1.85 m	2.55 m<br>2.28 m	2.58 m<br>2.33 m	1.61 m<br>1.45 m
3	3.41 t (3.2)			4.60 t (2.8)
4	-	-	-	-
5	1.60 m	1.39 m	1.32 m	1.30 m
6	1.47 m	1.51 m	2.02 m<br>1.25 m	1.41 m
7	1.43 m<br>1.15 m	1.33 m<br>1.11 m	1.45 m<br>1.20 m	1.39 m<br>1.18 m
8	-			-
9	2.07 m	1.47 m	1.33 m	1.37 m
10	-	-	-	-
11	1.67 m<br>1.49 m	1.95 m<br>1.21 m	1.45 m	1.99 m<br>1.15 m
12	1.81 m	1.84 m	1.75 m	1.79 m
13	2.04 m<br>1.18 m	1.65 m	1.53 m	1.93 m<br>1.56 m
14	1.89 m<br>0.78 m	1.90 m<br>0.84 m	1.88 m<br>0.84 m	1.85 m<br>0.78 m
15	1.12 s	1.18 m<br>1.09 m	1.49 m	1.10 s
16	-	-	-	-
17	3.48 d (11.6)<br>3.34 d (11.2)	3.49 d (11.2)<br>3.35 d (11.6)	3.97 d (10.0)<br>3.61 d (10.5)	3.47 d (11.6)<br>3.33 d (11.2)
18	0.85 s	1.27 s	1.07 s	0.91 s
19	0.97 s	1.27 s	1.04 s	0.84 s
20	1.00 s	1.14 s	1.10 s	1.02 s
21		4.09 q (7.2)	-	-
22		1.25 t (7.2)	1.38 s	2.03 s
23			1.37 s	

Table S2. ^{13}C-NMR data for compounds **1-4** in CD_3OD.

No	**1**	**2**	**3**	**4**
	δ_C ppm	δ_C ppm	δ_C ppm	δ_C ppm
1	73.6	35.1	38.1	34.0
2	30.3	30.1	34.3	24.4
3	78.8	177.0	217.4	80.0
4	38.8	76.1	47.8	37.6
5	44.5	53.5	55.8	51.8
6	19.4	23.4	23.6	19.3
7	40.5	40.0	39.1	40.8
8	34.0	34.1	33.4	34.0
9	44.0	45.1	50.6	53.0
10	42.8	42.3	37.4	38.6
11	24.5	23.9	19.8	24.1
12	33.1	35.5	34.2	33.1
13	23.3	24.2	23.5	23.4
14	28.6	27.9	27.3	28.3
15	54.1	53.3	54.1	53.6
16	75.2	75.0	82.8	75.1
17	69.7	69.7	74.6	69.7
18	22.3	28.4	26.4	22.2
19	29.1	32.7	21.8	28.6
20	15.1	19.0	13.8	14.4
21		61.4	109.0	172.6
22		14.6	27.6	21.1
23			27.6	

Table S3. Alpha-glucosidase inhibitory activity of **1-4**, **6**, and **7**.

**Compound**	**IC_{50} (μM)**
1	125.20
2	130.80
3	69.62
4	102.18
6	156.14
7	115.23
Acarbose	332.5

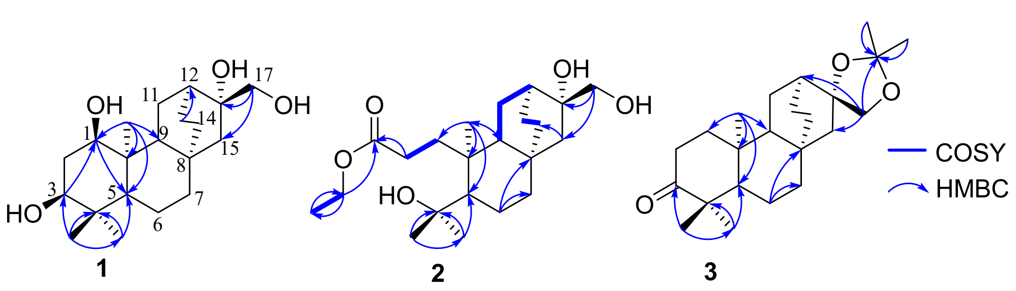

Figure S1. Key COSY and HMBC correlations of **1**-**3**.

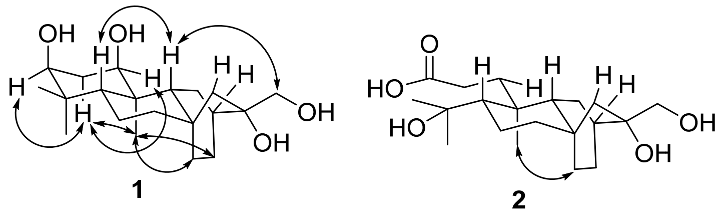

Figure S2. Key NOESY correlations of **1** and **2**.

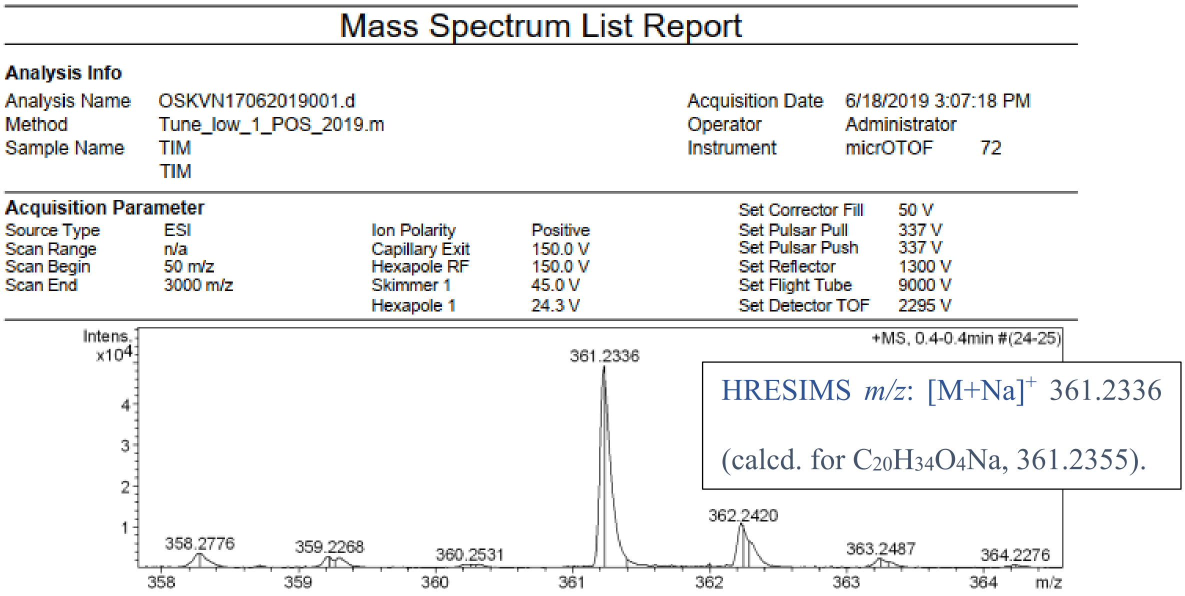

Figure S3. The HRESIMS spectrum of **1**.

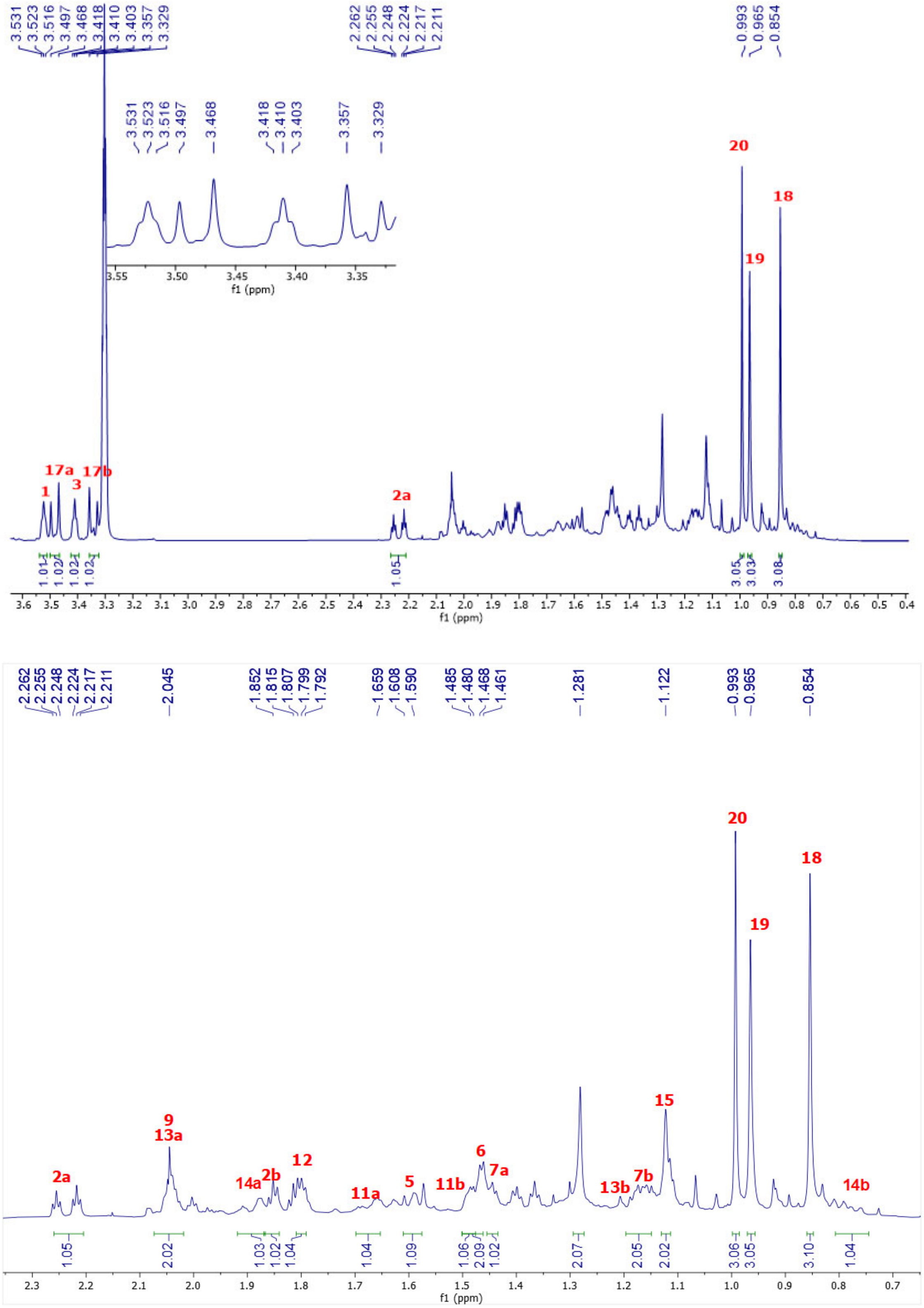

Figure S4. The ^{1}H NMR spectrum of **1** in CD_3OD.

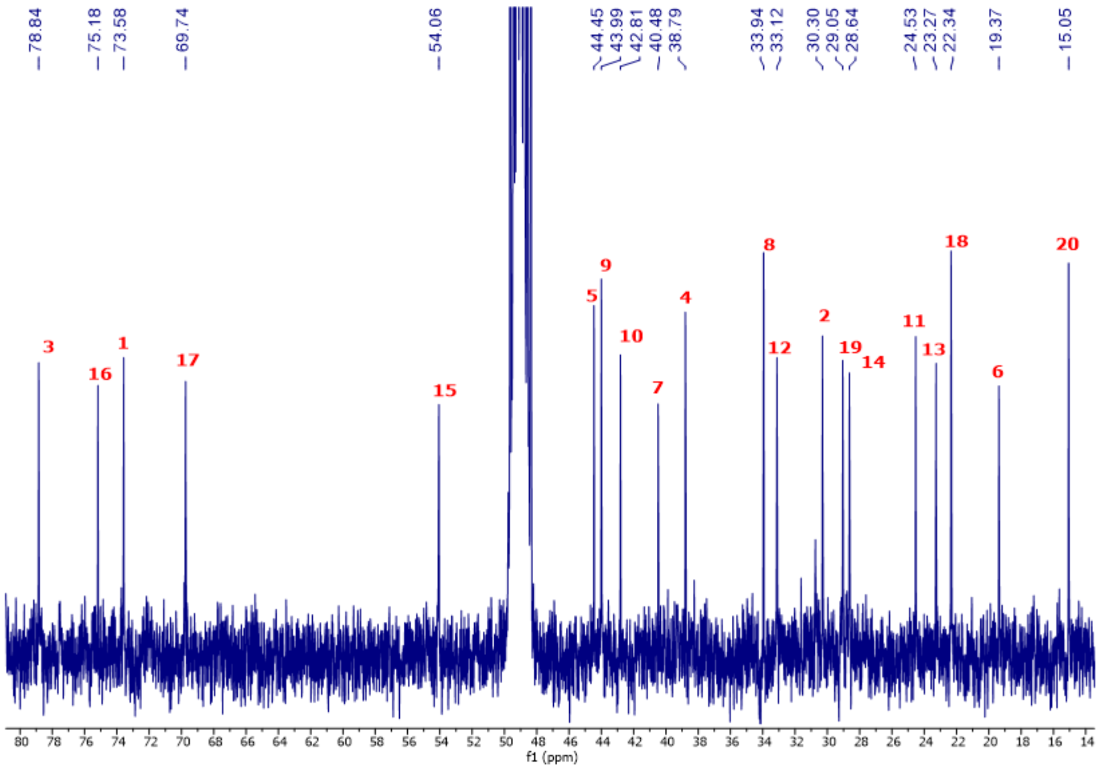

Figure S5. The ^{13}C NMR spectrum of **1** in CD_3OD.

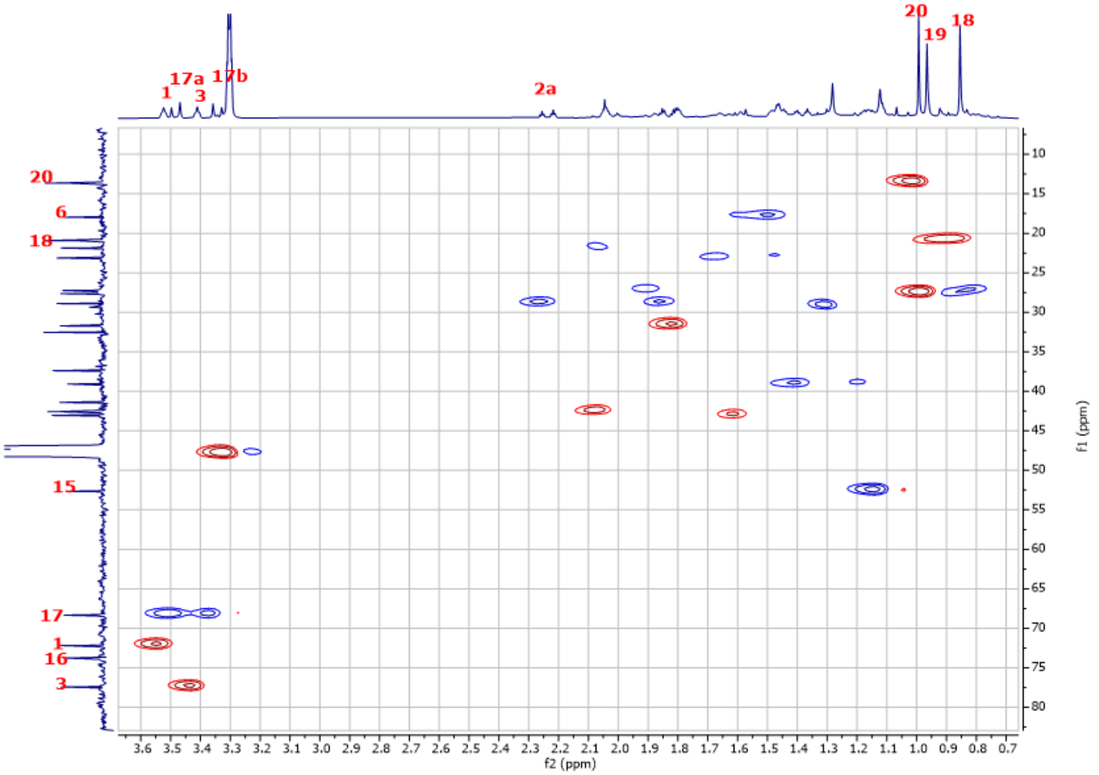

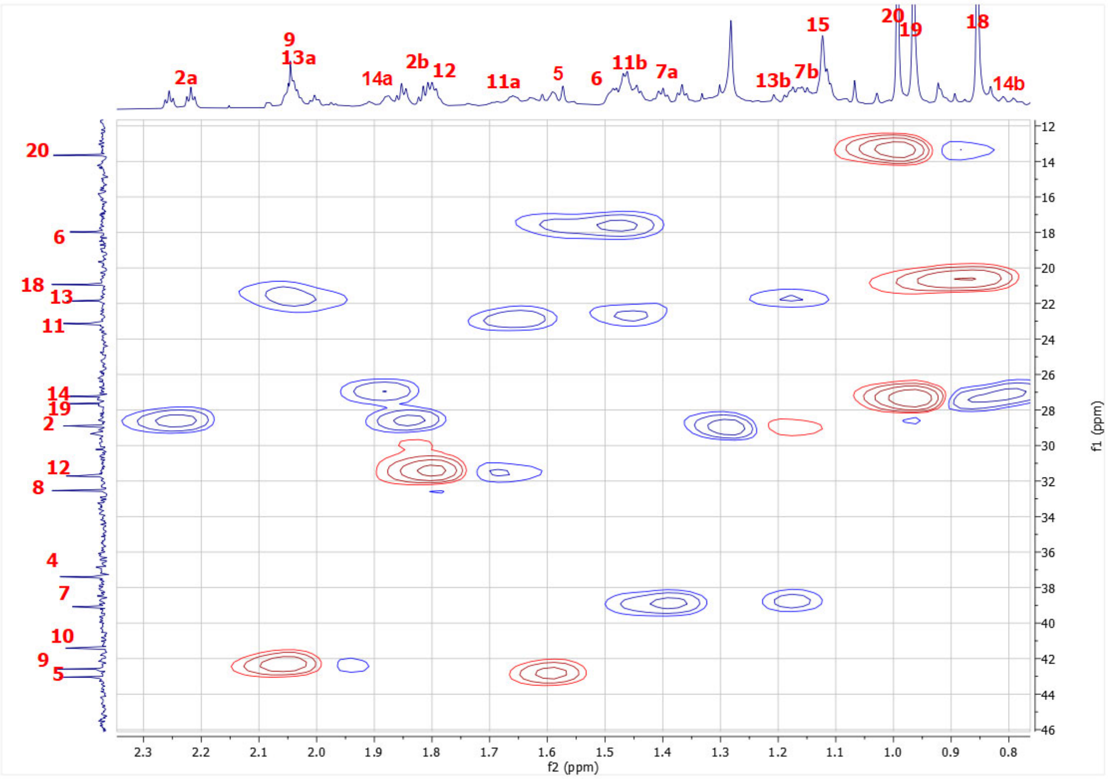

Figure S6. The HSQC spectrum of **1** in CD₃OD.

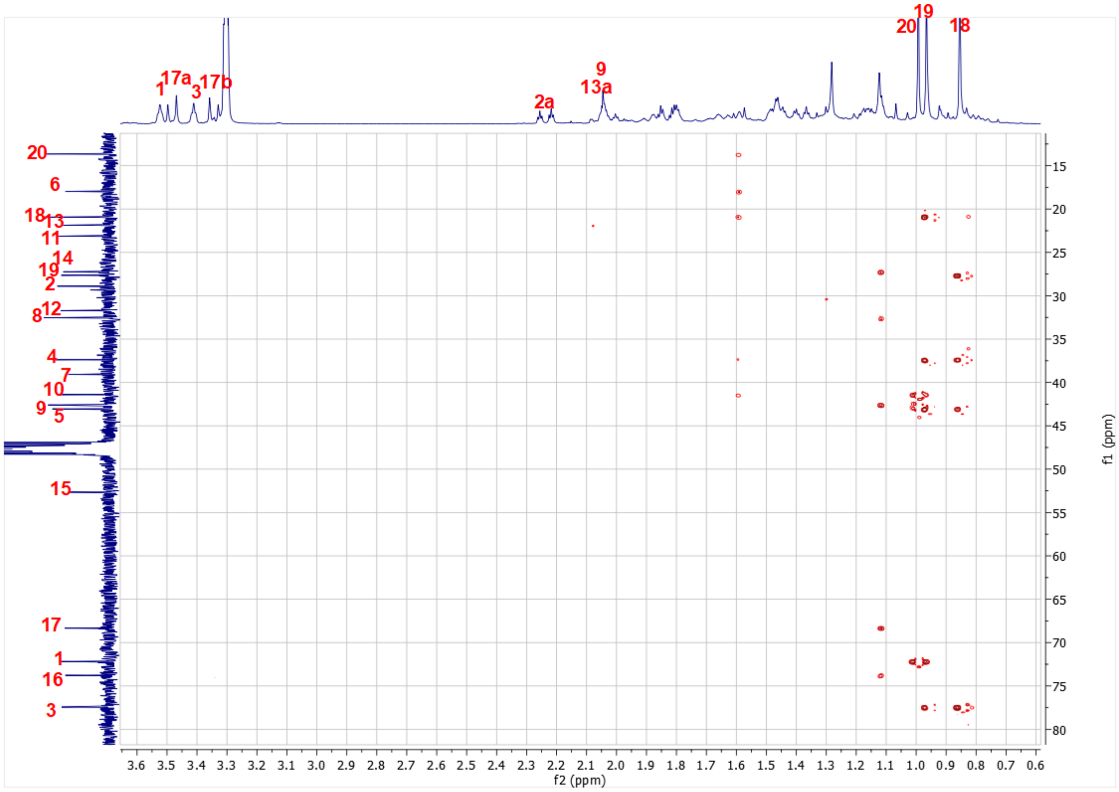

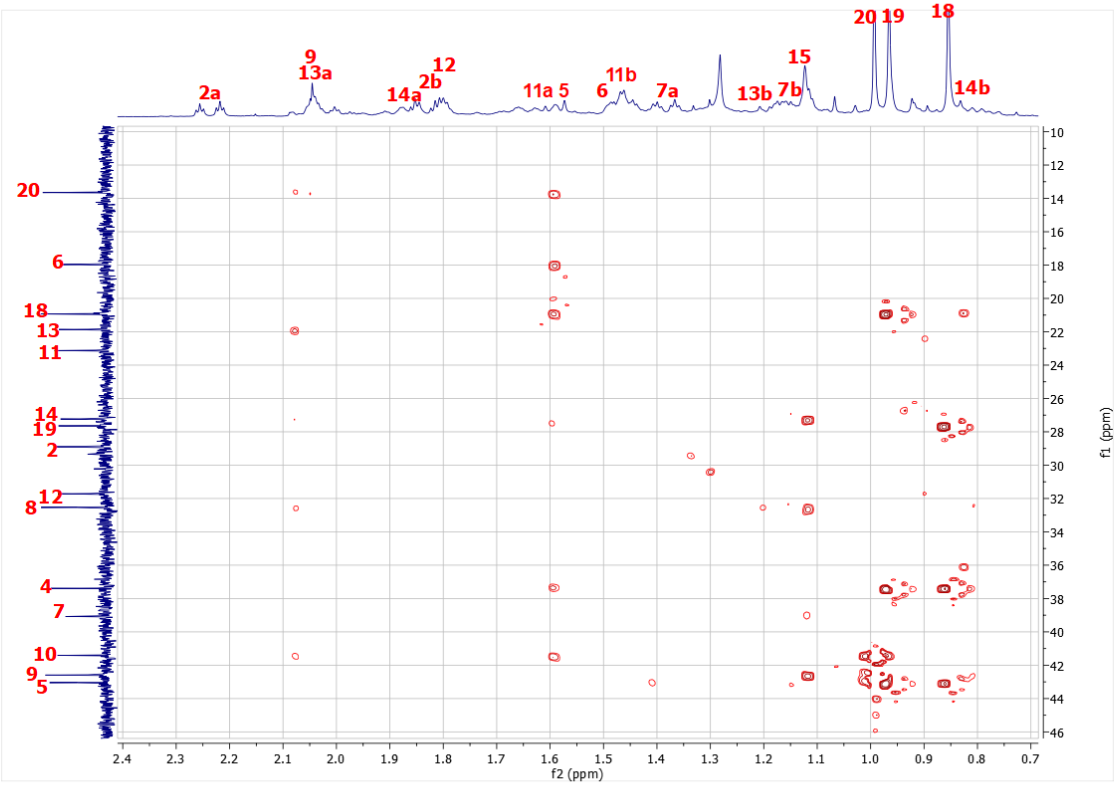

Figure S7. The HMBC spectrum of **1** in CD_3OD.

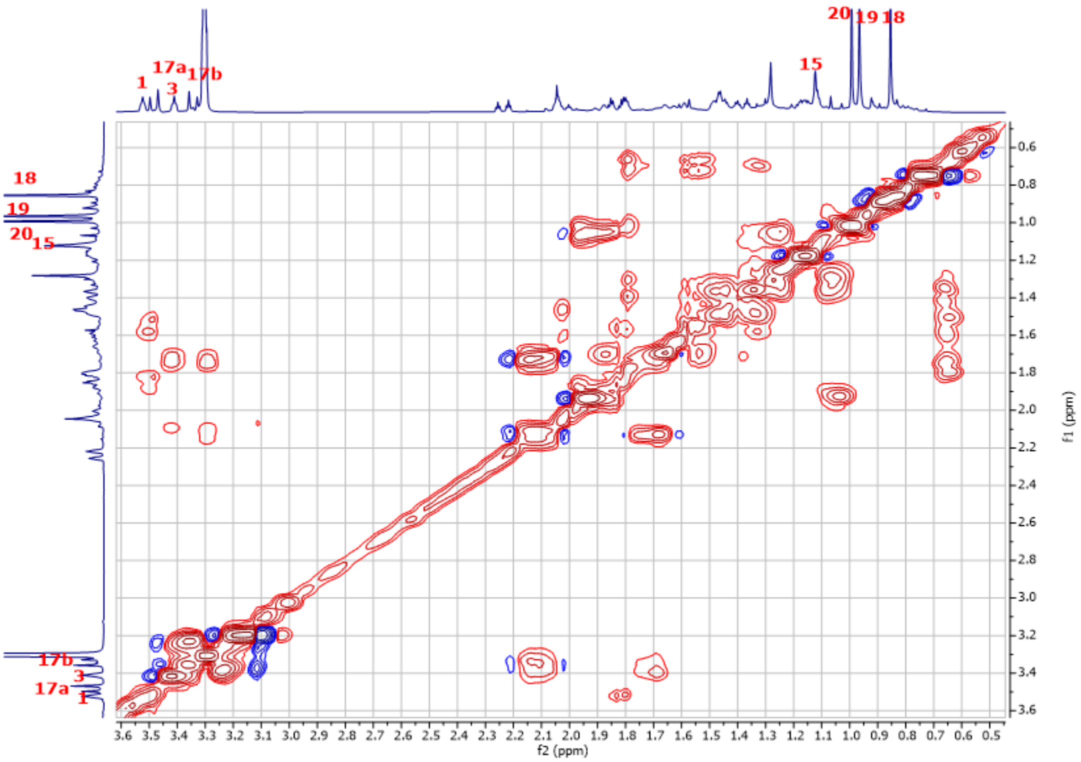

Figure S8. The COSY spectrum of **1** in CD_3OD.

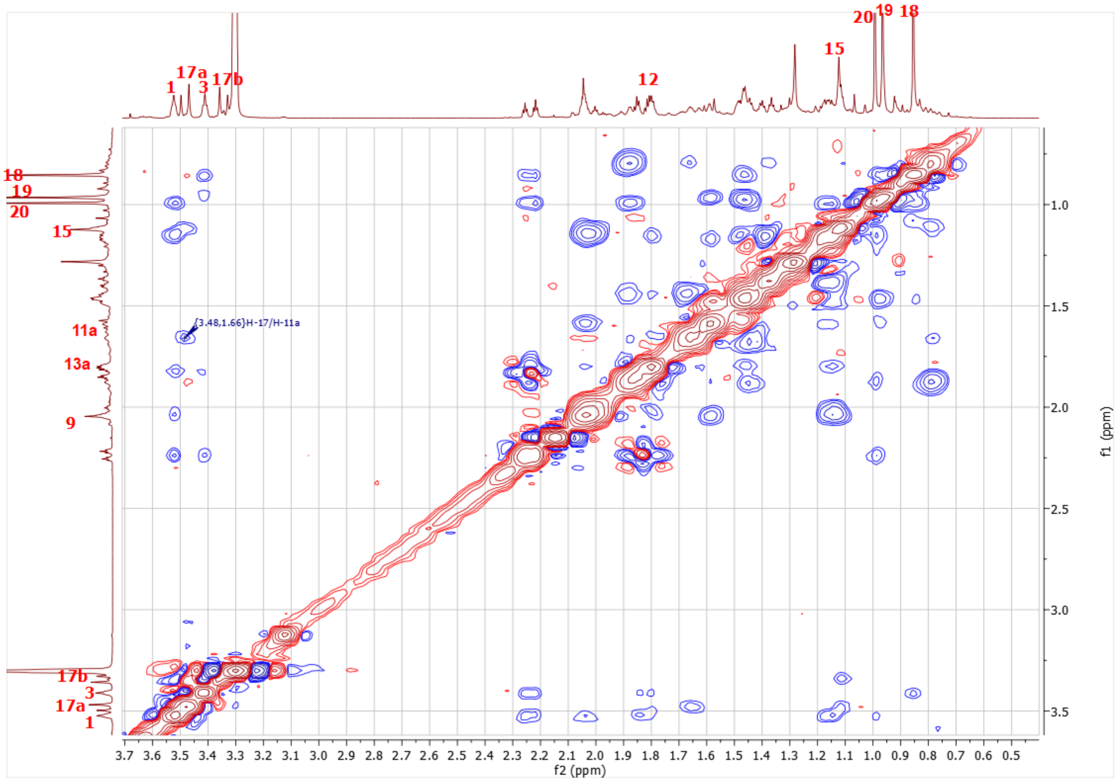

Figure S9. The NOESY spectrum of **1** in CD_3OD.

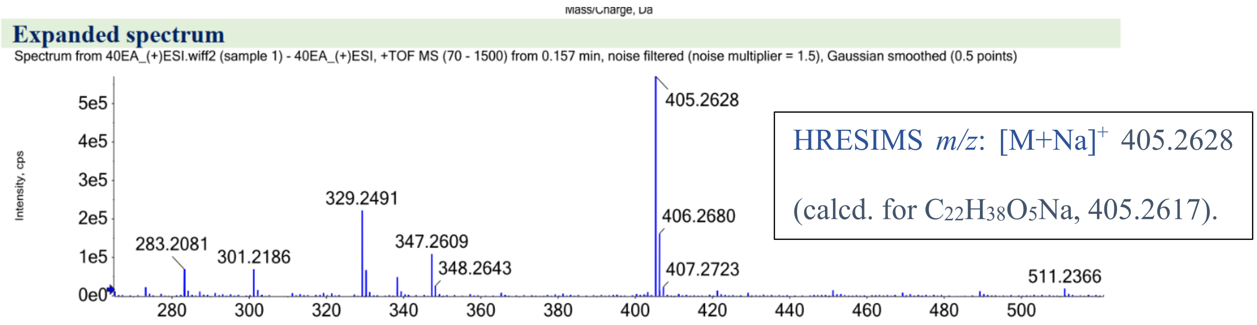

Figure S10. The HRESIMS spectrum of **2**.

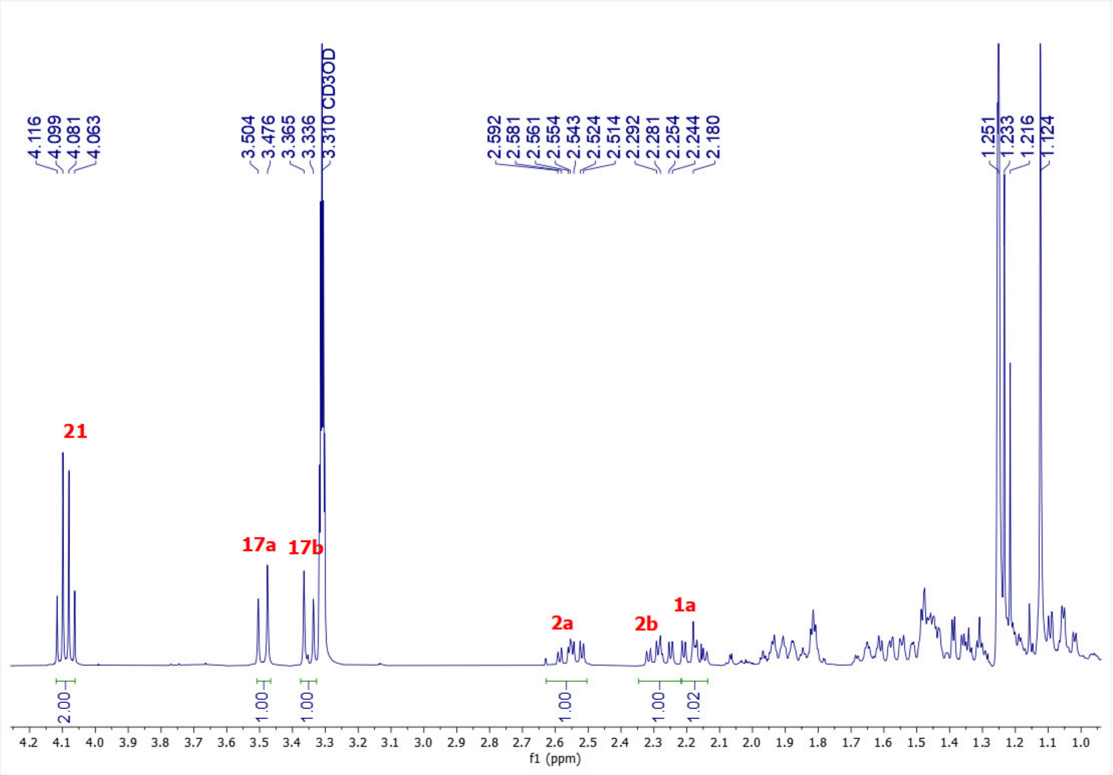

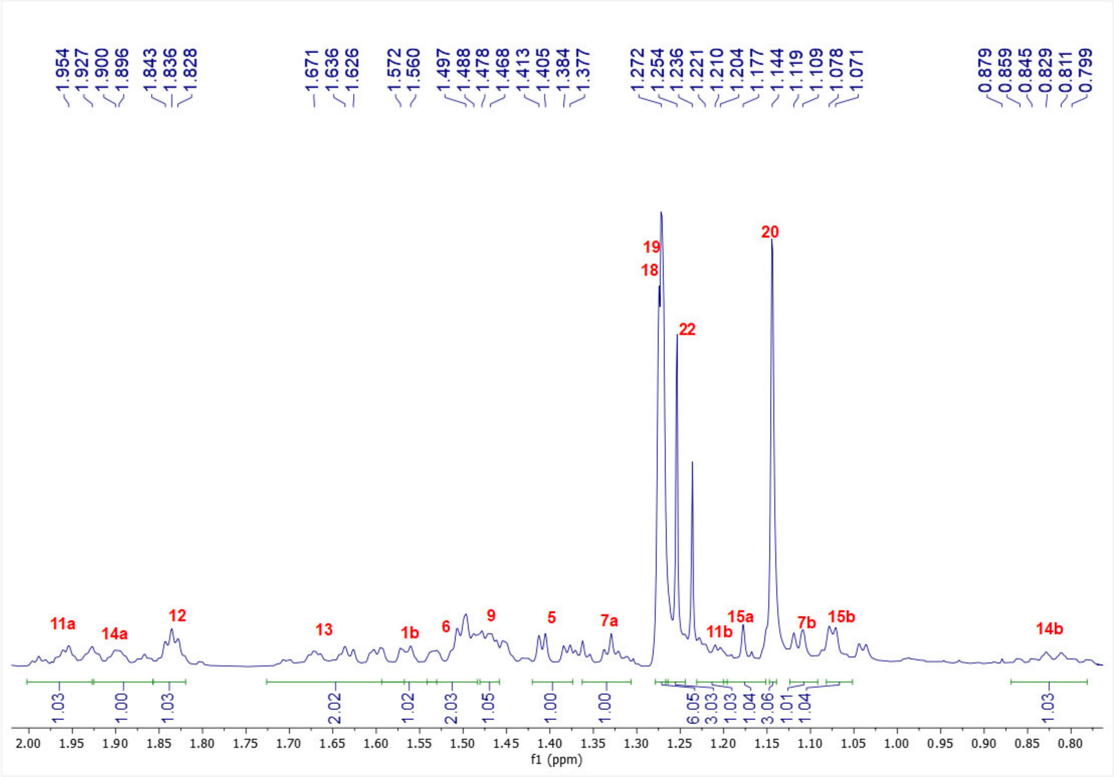

Figure S11. The ^{1}H NMR spectrum of **2** in CD_3OD.

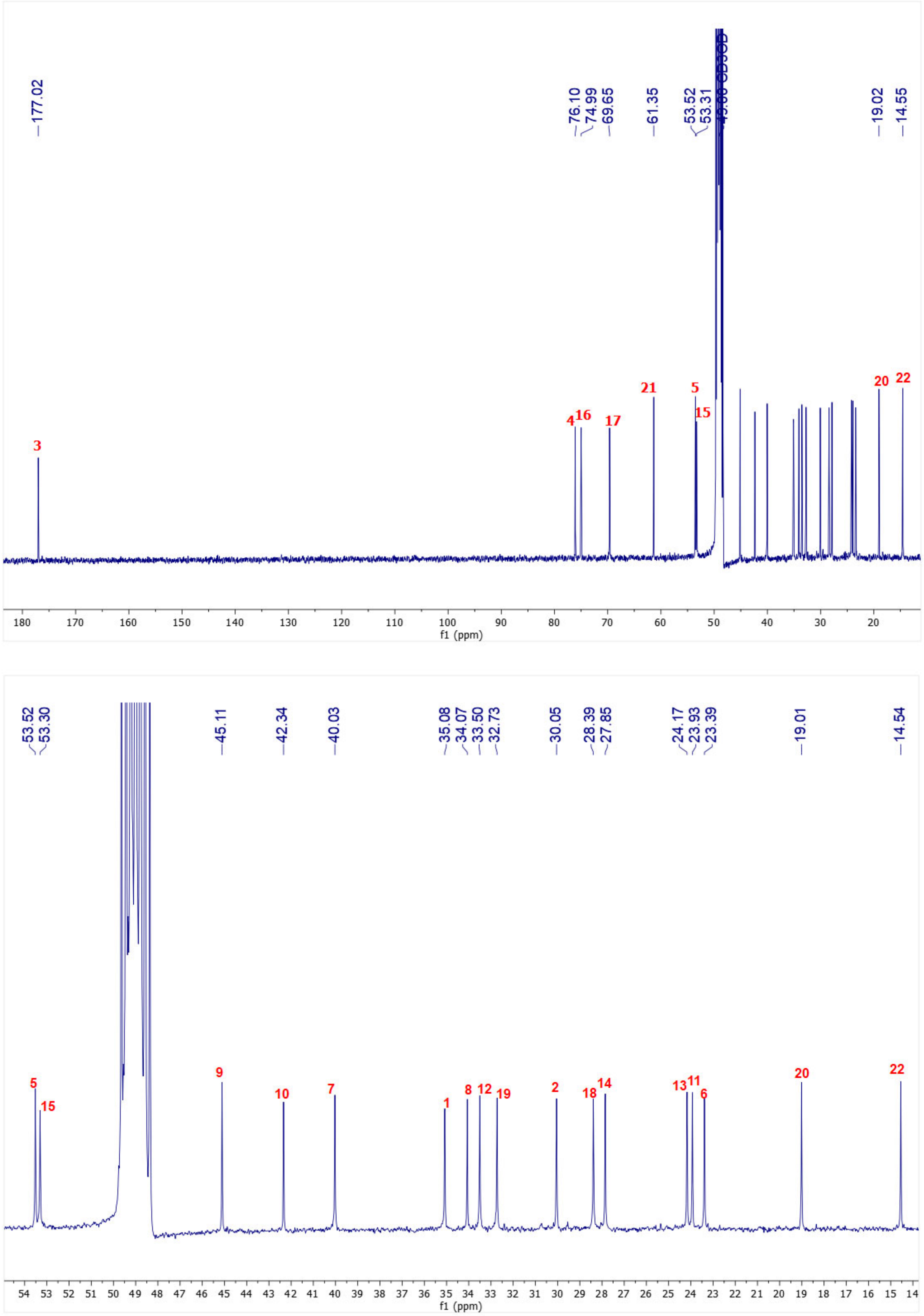

Figure S12. The ^{13}C NMR spectrum of **2** in CD_3OD.

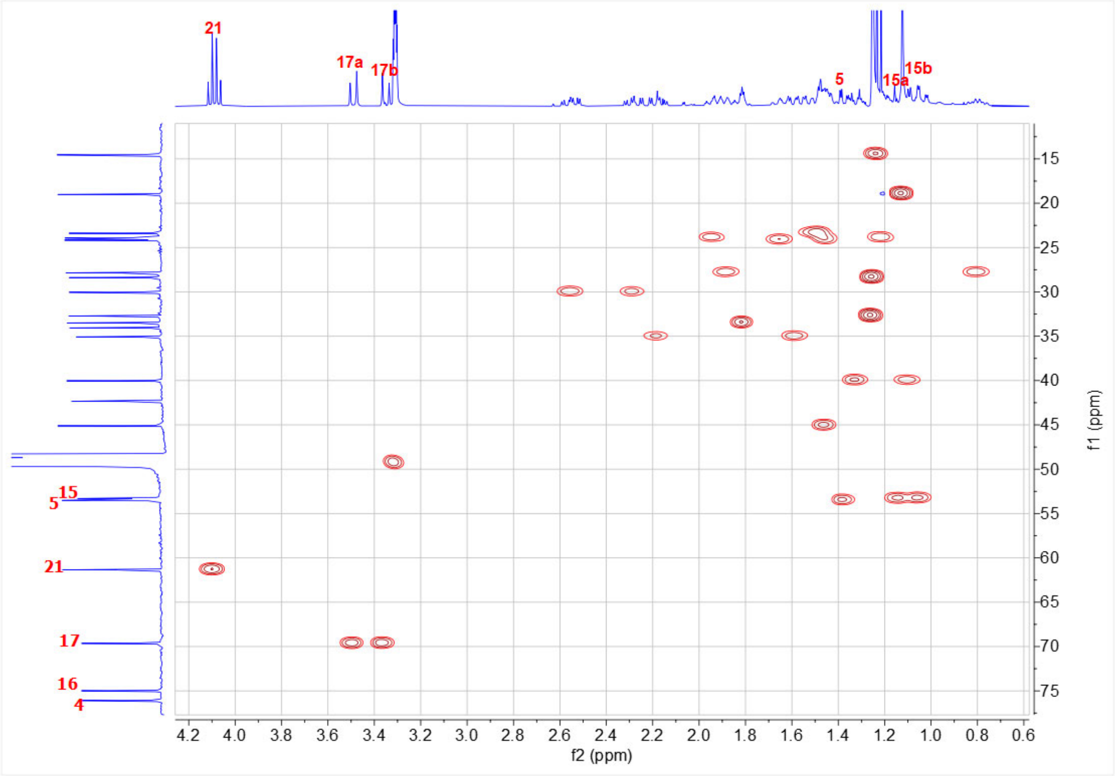

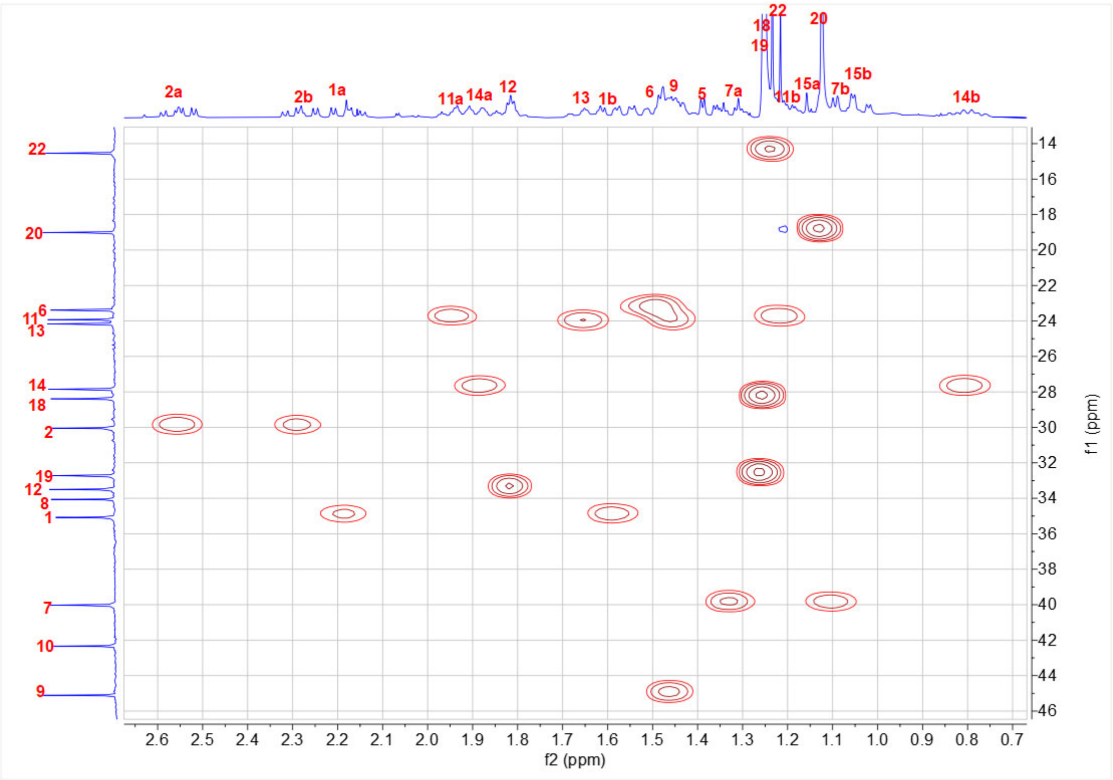

Figure S13. The HSQC spectrum of **2** in CD_3OD.

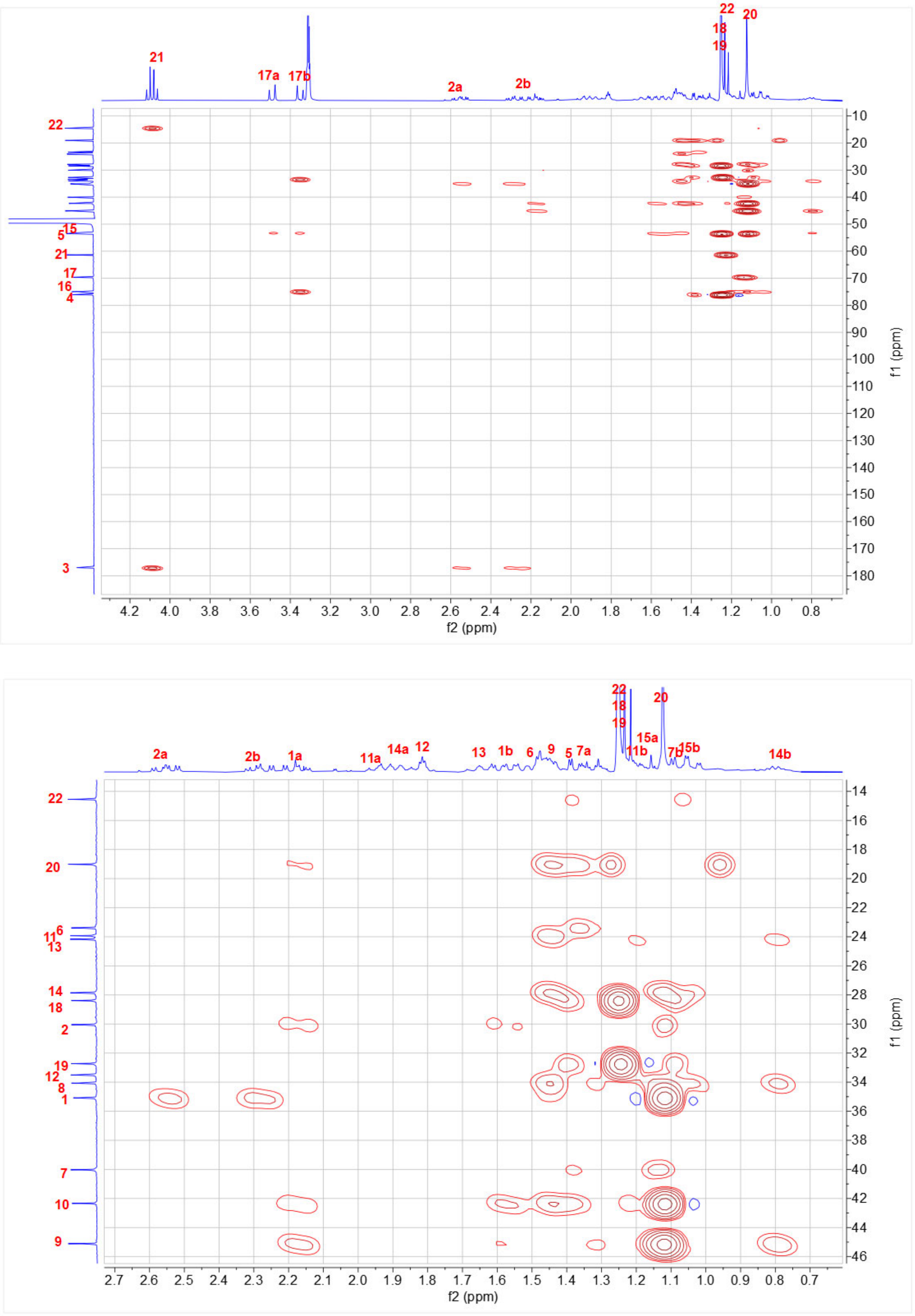

Figure S14. The HMBC spectrum of **2** in CD_3OD.

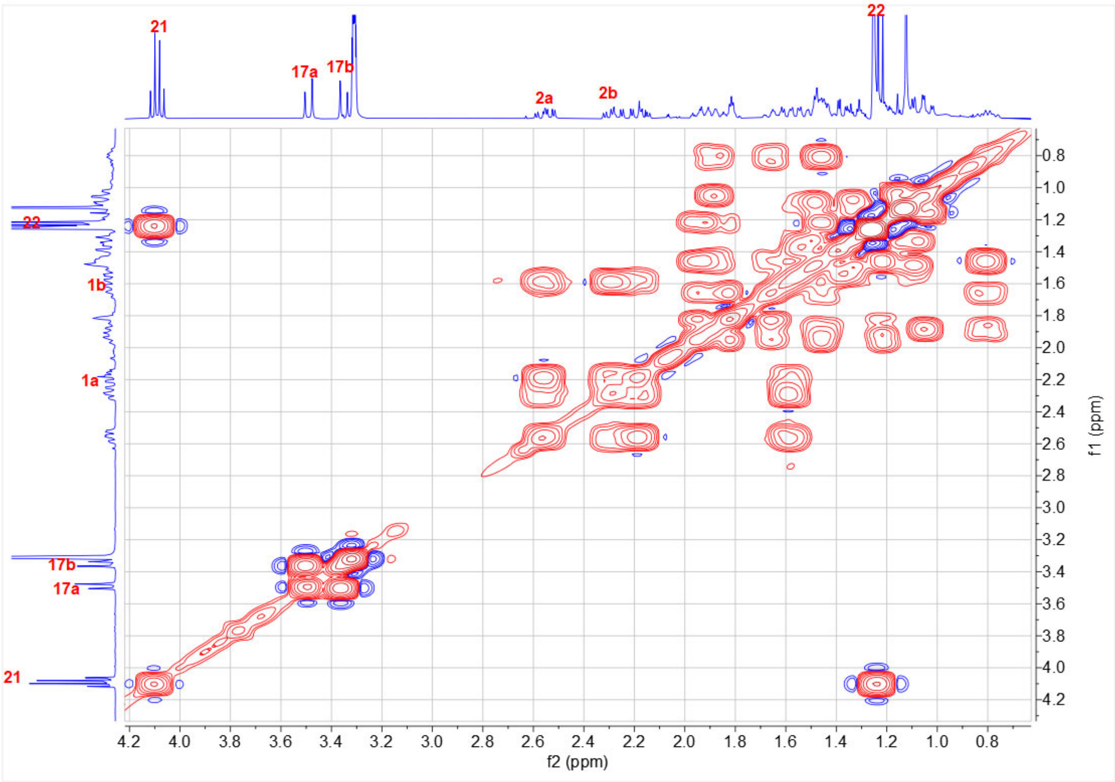

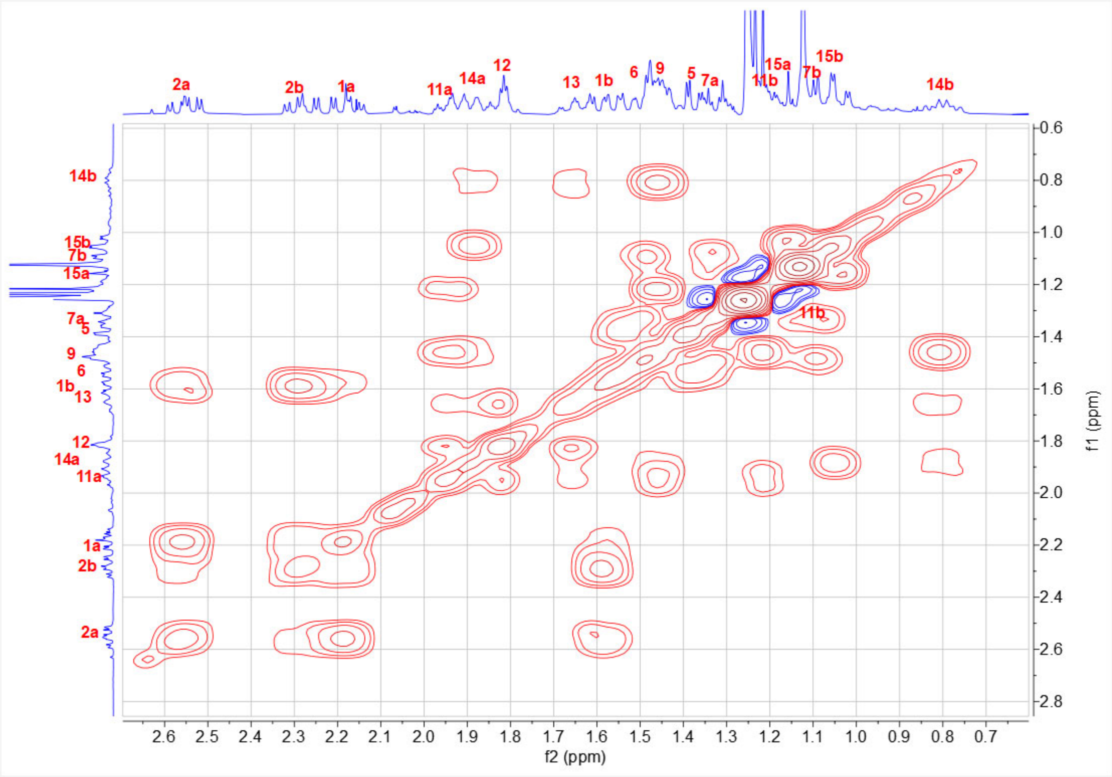

Figure S15. The COSY spectrum of **2** in CD_3OD.

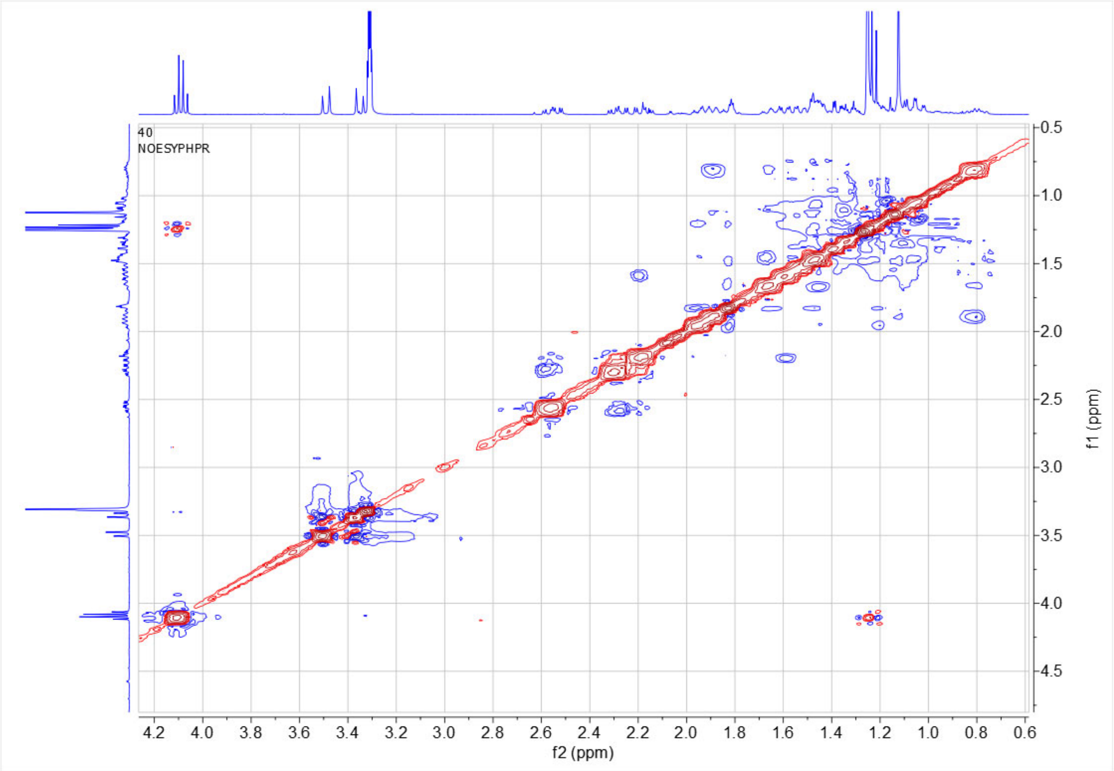

Figure S16. The NOESY spectrum of **2** in CD_3OD.

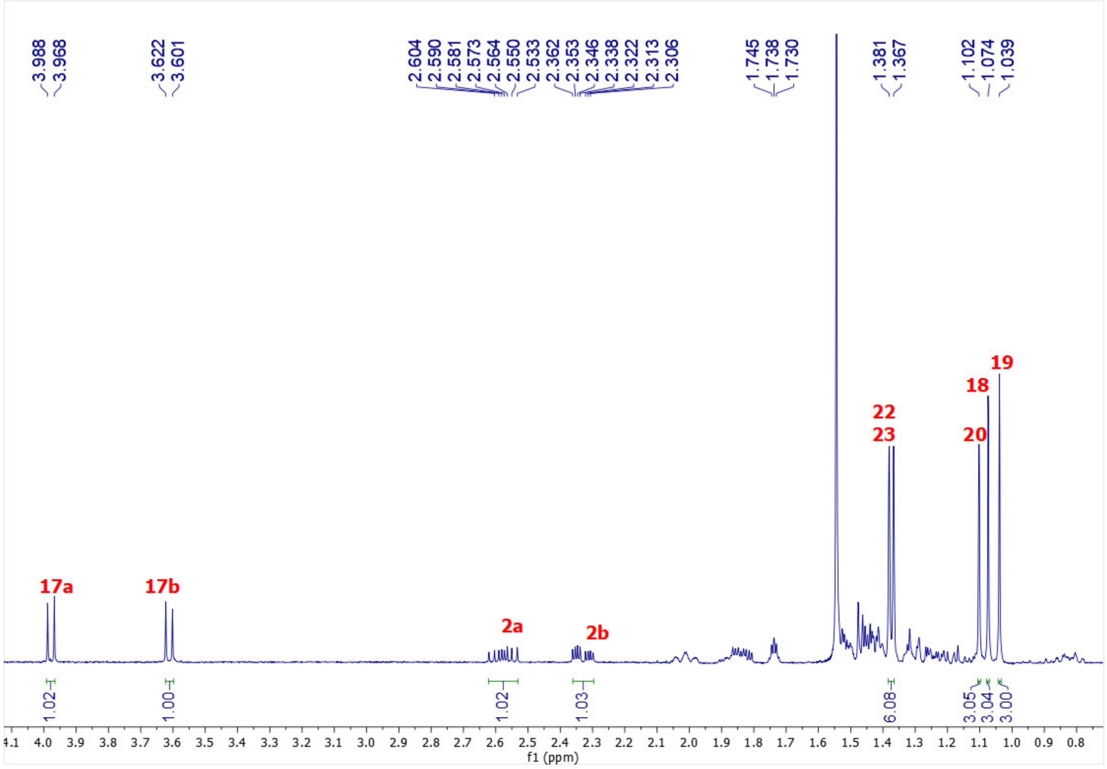

Figure S17. The ^{1}H NMR spectrum of **3** in CD_3OD.

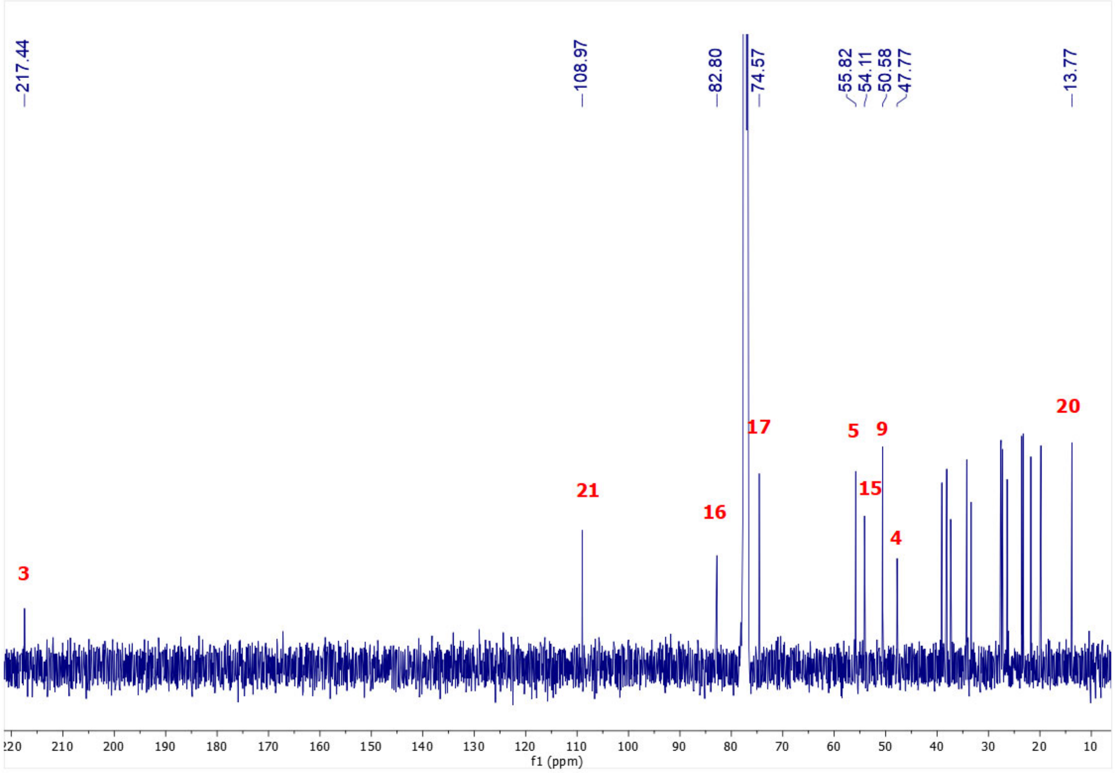

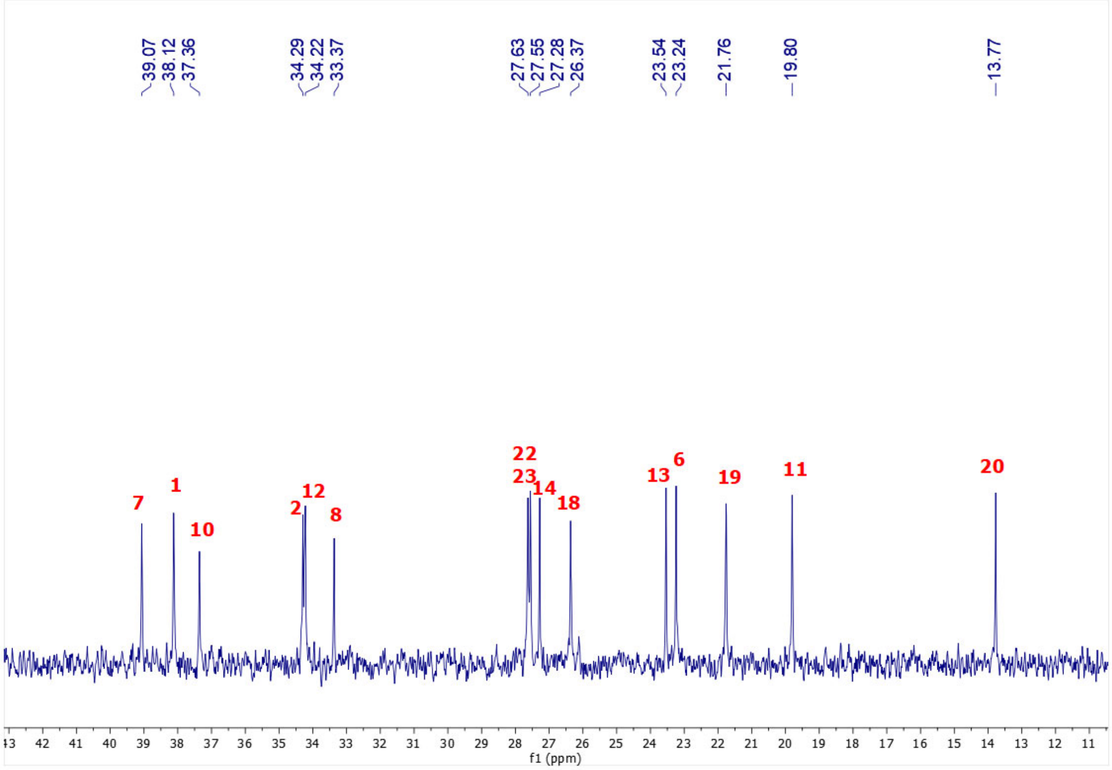

Figure S18. The ^{13}C NMR spectrum of **3** in CD_3OD.

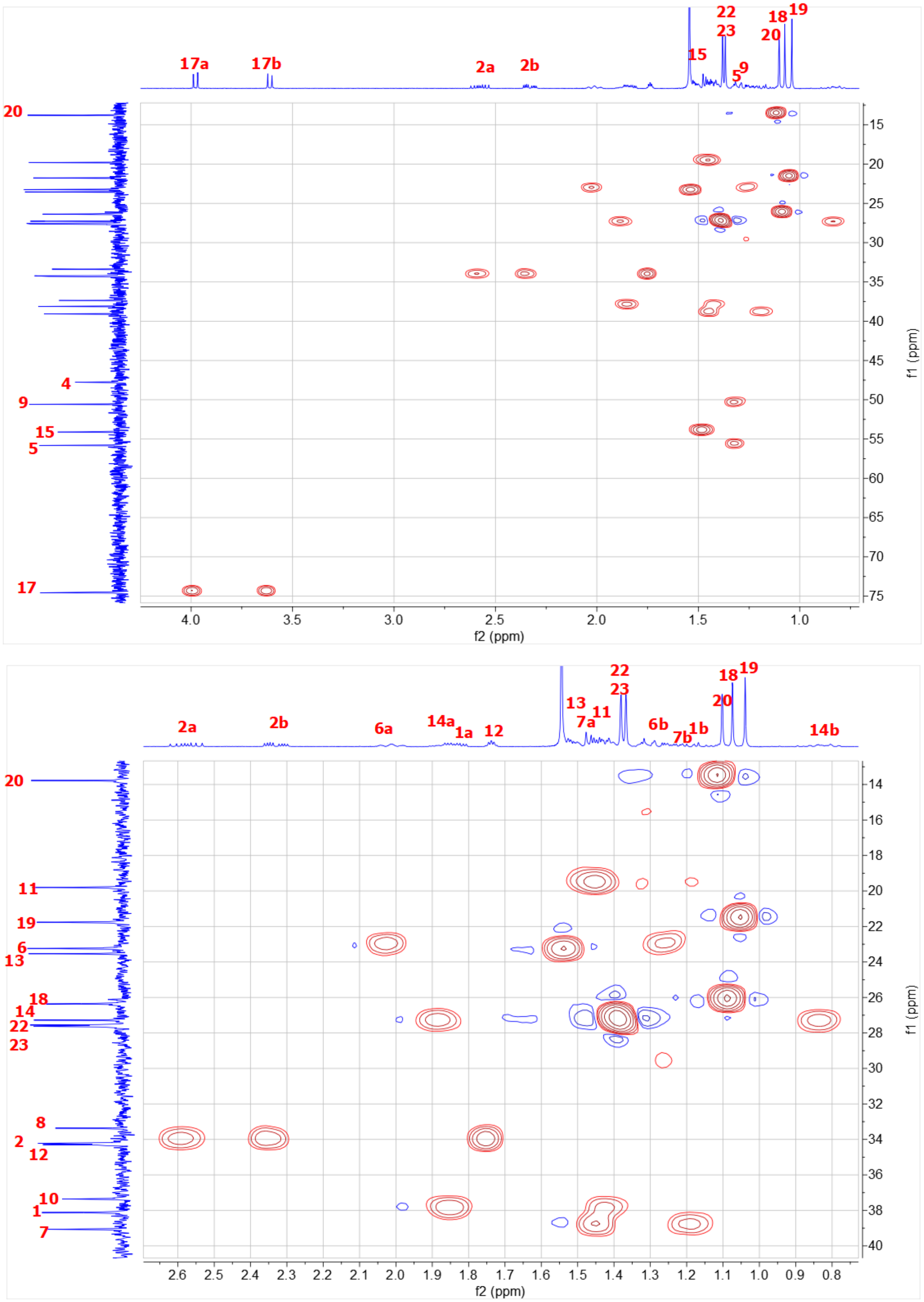

Figure S19. The HSQC spectrum of **3** in CD_3OD.

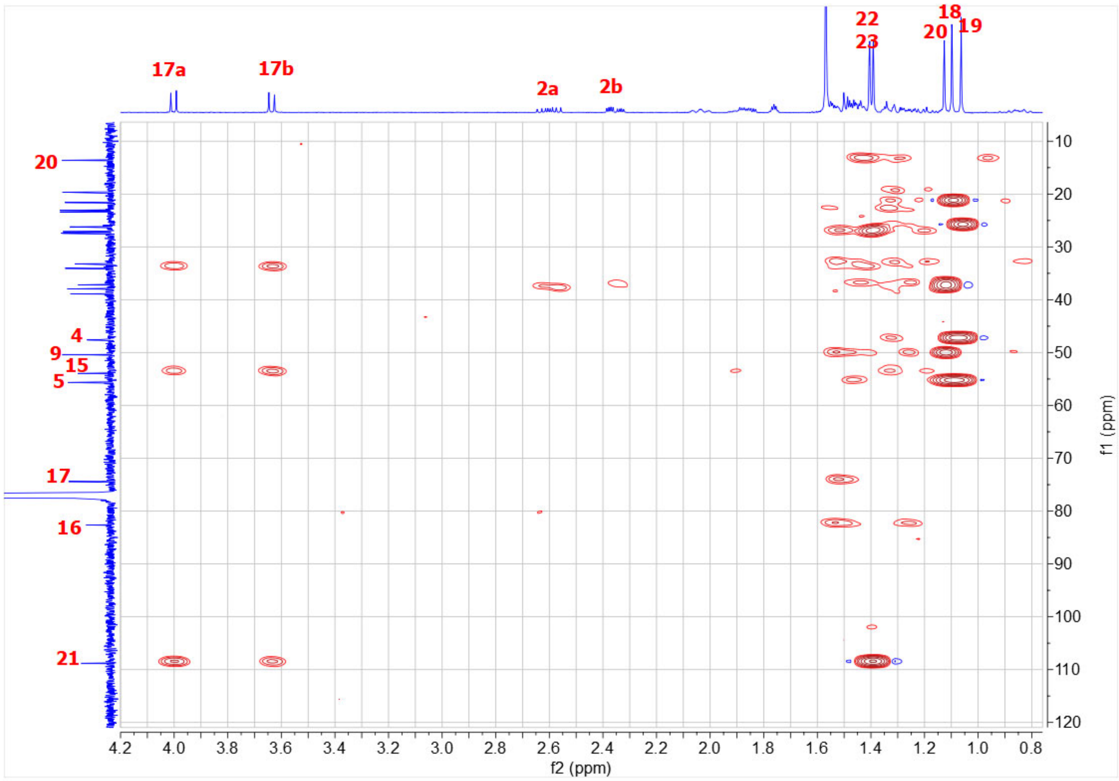

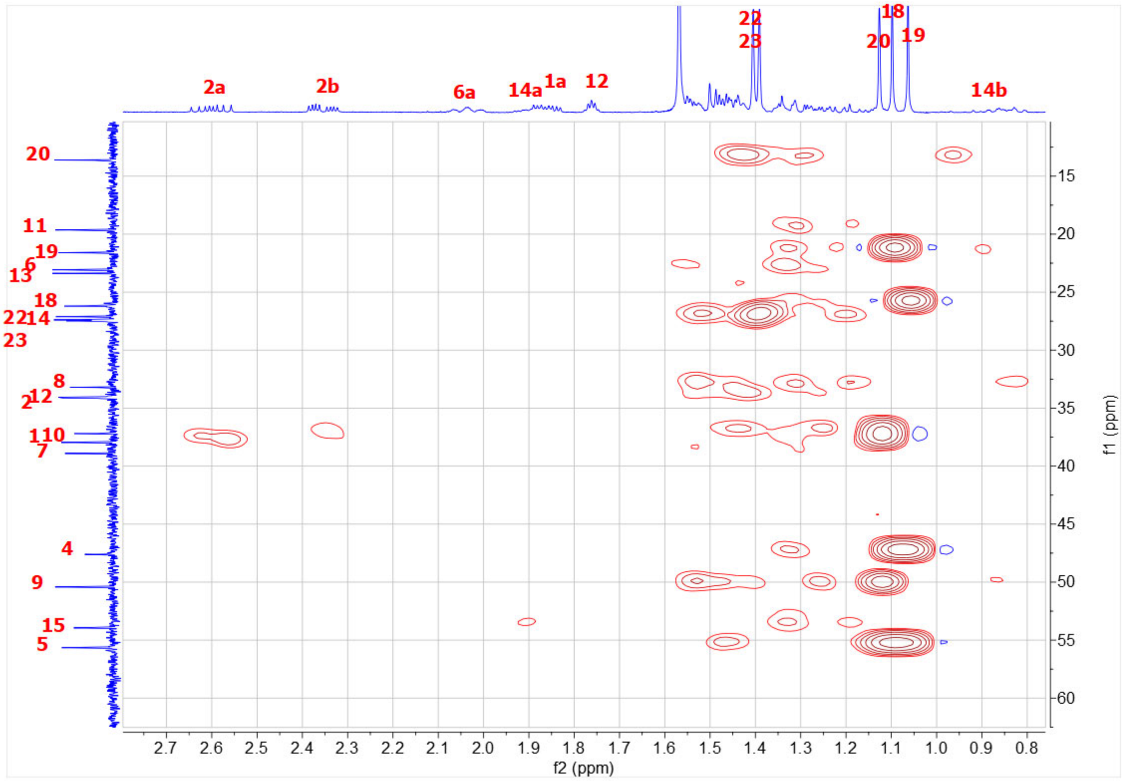

Figure S20. The HMBC spectrum of **3** in CD_3OD.

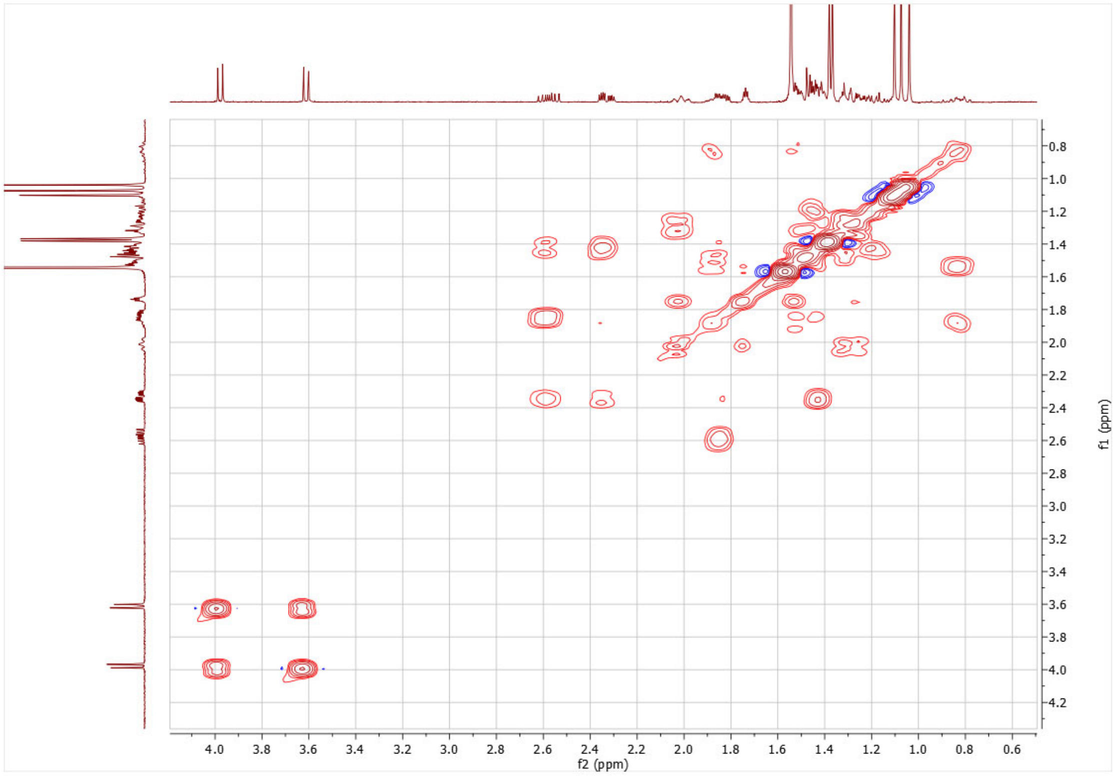

Figure S21. The COSY spectrum of **3** in CD_3OD.
